# Neuroimaging biomarkers in the biological definition of Parkinson’s disease and dementia with Lewy bodies – EANM position on current state, unmet needs and future perspectives

**DOI:** 10.1007/s00259-024-06803-w

**Published:** 2024-06-22

**Authors:** Matthias Brendel, Eric Guedj, Igor Yakushev, Silvia Morbelli, Günter U. Höglinger, Nelleke Tolboom, Antoine Verger, Nathalie L. Albert, Diego Cecchin, Pablo Aguiar Fernandez, Francesco Fraioli, Tatjana Traub-Weidinger, Donatienne Van Weehaeghe, Henryk Barthel

**Affiliations:** 1 Department of Nuclear Medicine, LMU Hospital, Ludwig-Maximilians-University of Munich, Marchioninstraße 15, 81377 Munich, Germany; 2 German Center for Neurodegenerative Diseases (DZNE), Munich, Germany; 3 Munich Cluster for Systems Neurology (SyNergy), Munich, Germany; 4 Département de Médecine Nucléaire, Aix Marseille Univ, APHM, CNRS, Centrale Marseille, Institut Fresnel, Hôpital de La Timone, CERIMED, Marseille, France; 5 Department of Nuclear Medicine, School of Medicine, Klinikum rechts der Isar, Technical University of Munich, Munich, Germany; 6 Nuclear Medicine Unit, AOU Città Della Salute E Della Scienza Di Torino, Turin, Italy; 7 Department of Medical Sciences, University of Turin, Turin, Italy; 8 Department of Neurology, LMU Hospital, Ludwig-Maximilians-University of Munich, Munich, Germany; 9 Department of Radiology and Nuclear Medicine, University Medical Centre Utrecht, Utrecht, The Netherlands; 10 Department of Nuclear Medicine and Nancyclotep Imaging, Platform, CHRU Nancy, Université de Lorraine, IADI, INSERM U1254, Allée du Morvan, 54500 Nancy, France; 11 Department of Medicine, Unit of Nuclear Medicine, University Hospital of Padova, Padua, Italy; 12 CIMUS, Universidade Santiago de Compostela & Nuclear Medicine Department, Univ. Hospital IDIS, Santiago de Compostela, Spain; 13 Institute of Nuclear Medicine, University College London (UCL), London, UK; 14 Department of Diagnostic and Therapeutic Nuclear Medicine, Clinic Donaustadt, Vienna Health Care Group, Vienna, Austria; 15 Department of Radiology and Nuclear Medicine, Ghent University Hospital, C. Heymanslaan 10, 9000 Ghent, Belgium; 16 Department of Nuclear Medicine, University Hospital Leipzig, Leipzig, Germany

## Introduction

Very recent publications proposed i) the three component system SyNeurGe [1] and ii) the neuronal α-synuclein disease integrated staging system (NSD-ISS) [2], both being based on a biological, biomarker-driven, definition of Parkinson’s disease (PD) and dementia with Lewy bodies (DLB). These definitions follow the biomarker-based ATN staging system of Alzheimer’s disease (AD) that includes biomarkers of beta-amyloid (A), tau (T), and neurodegeneration (N). As such, they represent a cornerstone for the fields and a huge step forward. The biological definitions of α-synuclein diseases were mainly motivated by the recent introduction of α-synuclein seed amplification assays that allow assessment of the neuropathological defining hallmark in vivo [3]. Accordingly, both frameworks envision the demonstration of α-synucleinopathy in the cerebrospinal fluid and/or skin. Of note, both research frameworks also include assessment of neurodegeneration and/or dopaminergic deficits via imaging biomarkers as complementary fundamental pillars in the biological definition along with genetics. The purpose of this editorial is to summarize the current status of molecular imaging methods, the anticipated impact and the unmet needs which require consideration in the molecular neuroimaging community in the context of biological definition of PD and DLB (Table 1 provides an overview). In this present article, we do not discuss reimbursement, due to complexity across health insurance systems in different countries.

**Table 1:** Summary of imaging methods discussed by the novel biological definition schemes of Parkinson’s disease

	Visual assessment of abnormality	Semi-quantitative assessment of abnormality	Procedure guideline	EMA/FDA approval
Definition of α-synuclein neuropathology (S+/S−)				
aSyn-PET	NA	NA	No	No
Definition of neurodegeneration and dopaminergic loss (N+/N− and D+/D−)				
Dopaminergic imaging (brain)	Yes	• Specific binding ratio (SBR) calculated as the striatal target-to-background ratio	Yes [9]	Yes
MIBG (heart)	No	• Heart to mediastinum ratio	No	No dedicated indication for PD and DLB
FDG-PET (brain)	Yes	• Regional standardized uptake value ratios • Pattern expression scores [23]	Yes [22]	No formal approval

## Definition of α-synuclein neuropathology (S+/S−)

In vivo assessment of underlying α-synuclein neuropathology that also defines PD and DLB post mortem is currently reserved for α-synuclein seed aggregation assays in cerebrospinal fluid or skin as well as immunohistochemical methods for skin. In this regard, PET imaging biomarkers for α-synuclein are on the horizon [4–6], but not yet validated or approved by regulatory authorities. Upcoming tasks for the molecular imaging community will consist in the participation in clinical phase 2 and phase 3 trials including autopsy validation with these novel α-synuclein PET ligands. Critically, similar to amyloid and tau PET imaging in AD, α-synuclein PET could refine in vivo staging via regional assessment of α-synuclein aggregation and facilitate development of disease-modifying therapies.

## Definition of neurodegeneration and dopaminergic loss (N+/N− and D+/D−)

**Fig. 1:**
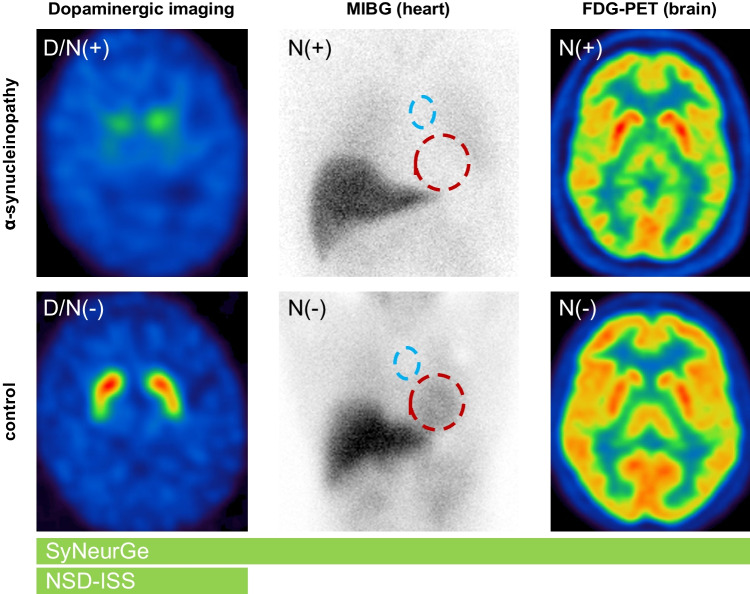
Schematic overview on imaging biomarkers of the dopaminergic system and neurodegeneration in PD and DLB as proposed by SyNeurGe and NSD-ISS

SynNeurGe includes three different imaging biomarkers for neurodegeneration, 2-[^18^F]fluoro-2-deoxy-D-glucose PET (FDG-PET) for assessment of cerebral glucose consumption patterns, dopaminergic imaging (i.e. with SPECT or PET) to detect dysfunction of nigrostriatal dopaminergic neurons and cardiac [^123^I]-meta-iodobenzylguanidine (MIBG) for assessment of sympathetic degeneration at post-ganglionic level (Fig. 1). Contrary, NSD-ISS includes DaT-SPECT as one pillar of the staging scheme (D+/D−) though promoting biomarkers of neurodegeneration only as an outlook (Fig. 1).

**Dopaminergic imaging** is possible by using several SPECT and PET tracers. [^123^I]ioflupane for SPECT is approved by the European Medicines Agency (EMA; EMA/267580/2011) and the US Food and Drug Administration (FDA; FDA/2011/022454) for diagnosis of clinically uncertain Parkinsonian syndromes and for differentiation between DLB from AD. Visual assessment is recommended, whereas semi-quantitative assessment is considered auxiliary. Here, thresholds used for assigning a pathological status are still under debate [7] and rely on different available quantification tools [8]. An updated EANM procedure guideline is available from 2020 [9]. Furthermore, several PET tracers for dopaminergic imaging that did not receive EMA or FDA approval yet are available, including [^18^F]FE-PE2I [10] and [^18^F]F-DOPA [11]. Despite staging of individual patients according to the degree of dopaminergic loss is in principle be possible, since quantitative measures correlate with the biological ground truth [12], reliable staging in centers with low experience is still limited. Harmonization of quantitative measures across cameras, reconstruction algorithms and methods (i.e. SPECT and PET), is an unmet need that should be addressed by the molecular imaging community. In this regard, there is emerging evidence that different thresholds should be used for the classification of an abnormal scan for PD and DLB patients [13]. This issue gets even more important when addressing prodromal stages, such as idiopathic/isolated REM sleep behaviour disorder (iRBD), where by modifying the abnormality threshold, the likelihood of phenoconversion prediction on a short term changes [14]. Accordingly, the clear definition of the DaT-SPECT abnormality threshold will have relevant clinical and research consequences, and efforts in harmonizing the assessment procedures and to identify disease- and stage-tailored cut-offs are urgently needed both for clinical practice and in disease-modifying trials. Centiloid units of amyloid PET imaging could serve as a successful template [15]. Moreover, the thresholds of abnormality should be revisited by analysis of large global databases. Here, the neuroimaging community should foster and lead global approaches using big data.

**[**^**123**^**I]MIBG** is FDA-approved (FDA/2008/22290) for detection of primary or metastatic pheochromocytoma or neuroblastoma, with a label extension in 2013 to assess myocardial sympathetic innervation in the evaluation of patients with NYHA class 2–3 heart failure with an LVEF < 35% [16, 17]. As such, its application is performed without dedicated indication for patients with Parkinsonian syndromes in several European countries. Due to the proposed value of MIBG in α-synucleinopathies, substantial numbers of patients have already been imaged and a large meta-analysis of 2680 subjects identified a heart-to-mediastinum ratio threshold of 1.77 to distinguish clinical α-synucleinopathies (excluding MSA) from disease controls and healthy controls [18]. However, no prospective validation or autopsy validation has been performed yet. First attempts of data harmonization across methodological setups have been successful [19], but inclusion of larger datasets is desired. The most urgent need in this context is to develop a procedure guideline for acquisition, reading and reporting of MIBG as an imaging tool to determine cardiac sympathetic denervation. Furthermore, the discussed biological definition of Parkinson’s disease should foster clinical trials that lead to MIBG label extension.

**[**^**18**^**F]FDG-PET** of the brain is frequently used in the diagnostic work-up of patients with suspected movement and cognitive disorders, but has no formal EMA or FDA approval for diagnosis of PD and DLB. This may be due to the absence of respective stakeholders due to the so-called ‘orphan’ drug status of this tracer preventing exclusive commercial rights for singular producers. As a consequence, rigorous expensive studies are scarce [20]. Nonetheless, FDG-PET is already considered a marker of neurodegeneration in the ATN system for AD [21]. An updated EANM procedure guideline on brain FDG-PET imaging is available from 2022 [22]. Topographic patterns of cortical and subcortical changes in glucose metabolism in suspected parkinsonian syndromes can be assessed by qualitative and univariate semi-quantitative methods. Furthermore, multivariate methods that enable to quantify expression of PD- and DLB-related patterns at the individual level have been proposed [23]. There are no licensed software packages, but available univariate solutions may be upgradable to quantification of PD- and DLB-related patterns. Similar to DaT-SPECT, quantitative pattern expression scores will require global harmonization. Upcoming tasks for the neuroimaging community consist in generating evidence for formal approval of FDG-PET by regulatory authorities and creation of unified databases for abnormality definition in the quantification of PD and DLB related patterns.

## Implications

Imaging parameters included in SyNeurGe and NSD-ISS can either be used as binary or as quantitative index to better describe disease state and stage of PD and DLB. Furthermore, imaging will enable to document different spatio-temporal processes such as brain-first versus body-first concepts. As imaging methods have been increasingly utilized for the biological definition of major neurological disorders such as AD (ATN) and PD/ DLB (SyNeurGe / NSD-ISS), health care systems, neurology and neuroimaging communities should discuss a cost-effective use and achieve reimbursement of these biomarkers. The understanding, implementation and utilization of nuclear medicine procedures and their interpretation (i.e. software) needs not only “imagers”, but also clinicians and support by industrial partners. Regardless, the newly proposed concepts of defining PD and related disorders mainly on biological grounds is a logical consequence of previous similar developments in the AD field, and of the recently introduced α-synuclein seed amplification assay technology. We welcome these concepts, since they pave the way for an earlier disease diagnosis allowing interventional studies in prodromal disease stages, and for a more stringent therapy monitoring in drug trials.

## Data Availability

Not applicable.
